# High Throughput Virtual Screening to Discover Inhibitors of the Main Protease of the Coronavirus SARS-CoV-2

**DOI:** 10.3390/molecules25143193

**Published:** 2020-07-13

**Authors:** Olujide O. Olubiyi, Maryam Olagunju, Monika Keutmann, Jennifer Loschwitz, Birgit Strodel

**Affiliations:** 1 Institute of Biological Information Processing: Structural Biochemistry, Forschungszentrum Jülich, 52428 Jülich, Germany; m.olagunju@fz-juelich.de (M.O.); Monika.Keutmann@uni-duesseldorf.de (M.K.); Jennifer.Loschwitz@uni-duesseldorf.de (J.L.); 2 Department of Pharmaceutical Chemistry, Faculty of Pharmacy, Obafemi Awolowo University, Ile-Ife 220005, Nigeria; 3 Institute of Theoretical and Computational Chemistry, Heinrich Heine University Düsseldorf, 40225 Düsseldorf, Germany

**Keywords:** COVID-19, docking, drug repurposing, natural products, in silico drug design, viral replication inhibition

## Abstract

We use state-of-the-art computer-aided drug design (CADD) techniques to identify prospective inhibitors of the main protease enzyme, 3CL^pro^ of the severe acute respiratory syndrome coronavirus 2 (SARS-CoV-2) causing COVID-19. From our screening of over one million compounds including approved drugs, investigational drugs, natural products, and organic compounds, and a rescreening protocol incorporating enzyme dynamics via ensemble docking, we have been able to identify a range of prospective 3CL^pro^ inhibitors. Importantly, some of the identified compounds had previously been reported to exhibit inhibitory activities against the 3CL^pro^ enzyme of the closely related SARS-CoV virus. The top-ranking compounds are characterized by the presence of multiple bi- and monocyclic rings, many of them being heterocycles and aromatic, which are flexibly linked allowing the ligands to adapt to the geometry of the 3CL^pro^ substrate site and involve a high amount of functional groups enabling hydrogen bond formation with surrounding amino acid residues, including the catalytic dyad residues H41 and C145. Among the top binding compounds we identified several tyrosine kinase inhibitors, which include a bioflavonoid, the group of natural products that binds best to 3CL^pro^. Another class of compounds that decently binds to the SARS-CoV-2 main protease are steroid hormones, which thus may be endogenous inhibitors and might provide an explanation for the age-dependent severity of COVID-19. Many of the compounds identified by our work show a considerably stronger binding than found for reference compounds with in vitro demonstrated 3CL^pro^ inhibition and anticoronavirus activity. The compounds determined in this work thus represent a good starting point for the design of inhibitors of SARS-CoV-2 replication.

## 1. Introduction

The recently identified COVID-19 (coronavirus disease 2019) causing virus is a coronavirus belonging to a very diverse group of the family enveloped RNA viruses of *Coronaviridae* [1,2]. Coronaviruses have been reported in different animal hosts and have been implicated in various respiratory and enteric infections of epidemic and pandemic proportion [1,3,4]. One of them, the SARS-CoV, was identified as the cause of the 2003 severe acute respiratory syndrome (SARS), an epidemic of pneumonia that resulted in more than 800 deaths worldwide [5]. In 2013, another member of the coronavirus group was found responsible for the Middle East respiratory syndrome coronavirus (MERS-CoV), an infection characterized by acute pneumonia and renal failure and with a fifty percent mortality rate recorded in admitted patients [6,7]. HCoV-229E, HCoV-OC43, HCoV-NL63, and HCoV-HKU1 are other identified human coronaviruses whose effect on the respiratory system results in milder forms of common colds [8,9].

In late 2019, a previously unknown member of the *Coronaviridae* family was identified and implicated in a global epidemic of respiratory systems. On 11 March 2020, the World Health Organization (WHO) declared the outbreak a pandemic. As of 28 May 2020, there are almost 6 million confirmed cases globally [10], and the infection fatality rate is reported to be around 0.4 [11]. The virus causing COVID-19 has been named SARS-CoV-2, because its RNA genome is about 82% identical to SARS-CoV [12]. Upon infection, COVID-19 affects first the upper respiratory tract with symptoms ranging from dry non-productive cough to sore throat and fever. Subsequently the lower trees of the respiratory tract are affected. However, the illness can also cause malaise, confusion, dizziness, headaches, digestive issues, and a loss of smell and taste. It has been suggested that these neurological signs may result from the ability of the virus to invade the central nervous system [13]. With its highly effective mode of transmission, COVID-19, in spite of its relatively low fatality rate [11], represents one of the greatest public health challenges in recent times.

Unfortunately, there are currently no antiviral drugs or vaccines approved for COVID-19 or any other human coronavirus infections [9]. The genome of SARS-CoV-2 encodes for different proteins, including the 3-chymotrypsin-like protease (3CL^pro^), also called main protease (M^pro^), papain-like protease, helicase, and RNA-dependent RNA polymerase [14,15]. Since the main protease 3CL^pro^ is crucial for viral replication and well conserved across the *Coronaviridae* family, it represents a viable target for drug design [12]. 3CL^pro^ cleaves the large polyprotein 1ab (replicase 1ab, ∼790 kDa) at eleven or even more cleavage sites involving, in most cases, the recognition sequence L-N*(S,A,G) (* marks the cleavage site), yielding functional proteins that are then packed into the virion. Another advantage of targeting 3CL^pro^ is that although the mutagenesis rate is high in viruses, this does not apply to this protein since any mutation here can be fatal for the virus. Furthermore, since no human proteases with a similar cleavage specificity are known, it should be possible to identify inhibitors of no or low toxicity.

**Figure 1:**
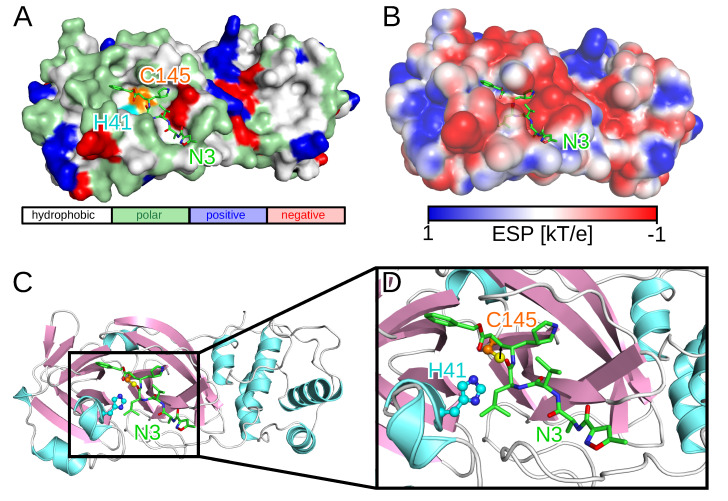
Structure and binding site of 3CL^pro^ including the ligand N3 (PDB code 6LU7). (**A**) The surface of 3CL^pro^ is shown and colored according to residue type using the color code given at the bottom. The catalytic dyad residues H41 and C145 are colored in cyan and orange, respectively. The stick representation is used for N3, which is colored in green with red and blue for the O and N atoms, respectively. (**B**) Another surface representation of 3CL^pro^ but now colored according to the electrostatic potential using the color code given at the bottom (in kT with *k* being the Boltzmann constant, created with the Adaptive Poisson–Boltzmann Solver (APBS) software [29,30]). (**C**) Cartoon representation of 3CL^pro^ with β-sheets shown in lilac and α-helices in light blue. The sidechains of H41 and C145 are shown in ball-and-stick representation in cyan and orange, respectively, but using blue for N atoms, red for O atoms, and yellow for the S atom of C145. (**D**) Zoom into the substrate binding site of 3CL^pro^. In panels B–D the same representation for N3 as in panel A is used.

Since the outbreak, several SARS-CoV-2 protein structures have been solved using either X-ray diffraction or cryo-electron microscopy. One of these structures, employed in this work, is the crystal structure of the SARS-CoV-2 3CL^pro^ enzyme in complex with a synthetic peptidomimetic inhibitor called N3 (PDB code 6LU7, Figure 1) [16]. While the catalytically active form of 3CL^pro^ is a dimer, the two protomers most likely act independently from each other as the two active sites are solvent-exposed and symmetrically located at opposite edges of the cleft between the two protomers [17]. The proteolytic process in the active site of 3CL^pro^ is enabled by the catalytic C145-H41 dyad with the cysteine thiol group acting as the nucleophile (Figure 1C) [18]. The initial drug discovery efforts after the SARS outbreak in 2003 aimed at electrophilic attack to the cysteine residue of the catalytic dyad via covalent Michael inhibitors [19]. While this was considered to be safe due to the different proteolytic cleavage specificities between SARS-CoV and human proteases, electrophiles are usually no good drug candidates as they often cause adverse effects such as allergies, tissue destruction, or carcinogenesis [20]. After 2005–2006, many of the initial efforts of developing small-molecule compounds with anticoronavirus activity were discontinued due to a sharp decline in funding of coronavirus research as it was erroneously assumed that another zoonotic coronavirus transmission was extremely unlikely to happen again. Thus, none of these attempts resulted in an anticoronavirus drug, not even the clinical stage was reached.

This drastically changed after the COVID-19 outbreak. Less than four months after the first cases were reported in Wuhan in China, several studies aiming at designing and developing treatment for the disease have already been published [16,21,22,23,24,25,26,27]. In one of these studies, the mechanism-based inhibitor N3 was designed (Figure 1) using computer-aided drug design (CADD) techniques [16]. Seven further compounds were identified in that study through a combination of structure-based virtual and high-throughput screening of over 10,000 compounds, including approved drugs, drug candidates in clinical trials, and other pharmacologically active compounds, as inhibitors of 3CL^pro^ with half maximal inhibitory concentrations (IC_50_) ranging from 0.67 to 21.4 μM. The strongest antiviral activity in cell-based assays was found for ebselen, a synthetic organoselenium drug molecule with anti-inflammatory, anti-oxidant and cytoprotective activity which is currently in a clinical trial as a potential treatment for bipolar disorder [28].

Here, we use high-throughput virtual screening to discover potential inhibitors of 3CL^pro^ of SARS- CoV-2. We screened over 1 million compounds covering multiple compound libraries most of which are available through the ZINC database [31,32,33], which is a curated collection of more than 230 million commercially available chemical compounds prepared for virtual screening. The screened compounds include drugs approved by the U.S. Food and Drug Administration (FDA) or other authorities, investigational drugs, natural products, as well as organic compounds that do not necessarily fall into the other mentioned libraries [34,35]. In addition, we also scanned our in-house database with almost 3200 natural compounds isolated from African plants. We employed a virtual screening against the crystallographic 3CL^pro^ structure as well as ensembles of the SARS-CoV-2 enzyme generated from an explicit-solvent molecular dynamics (MD) simulation. We present here our analyses and important findings relating to the inhibition of 3CL^pro^, which we believe can guide the search for a COVID-19 treatment.

## 2. Results and Discussion

In this work, we have employed CADD approaches in search of compounds that are capable of forming thermodynamically feasible binary complexes with the SARS-CoV-2 main protease enzyme 3CL^pro^. We adopted a protocol that is slightly different from previous works conducted by other groups in the search for potential inhibitors of this critical enzyme [16,21]. First, we have not limited our virtual screening to previously known inhibitors of viral proteases but instead embarked on what would count as an unbiased screening of a fairly large compound library. We believe this approach reduces the possibility of omitting potentially useful compounds, especially considering that the known inhibitors were tested on different viral species. Second, our screening procedure incorporated protein dynamics in order to model as accurately as possible the conformational preferences available in vitro and in vivo for substrate recognition. The immediate objective of our research work is the identification of small-molecule inhibitors of 3CL^pro^, and in particular of approved drugs which should reduce the amount of time to clinical readiness. This also holds true for natural products present in plants that are available as herbal, nutraceutical or food products.

Another objective is to determine the structural basis of inhibitor interaction with 3CL^pro^ and identify chemical structures capable of serving as templates for the design of potent inhibitors. We expect that the chances of finding suitable inhibitors will be enhanced by screening chemically diverse libraries. To this end, we screened a library of 1,227,186 compounds derived from the ZINC library [31,32,33] as well as our in-house library of natural products of Nigerian origin (unpublished) against the crystallographic structure of the functional form of the SARS-CoV-2 3CL^pro^ enzyme (PDB code 6LU7 [16]). We also included in our screening eight recently reported inhibitors of the SARS-CoV-2 3CL^pro^ enzyme, which are the peptidomimetic N3 present in the crystal structure used in this work as well as ebselen, disulfiram, carmofur, cinanserin, shikonin, tideglusib, and PX-12 reported alongside this crystal structure [16]. All eight reference compounds were able to bind to the active site of the crystal structure of the enzyme. The best binding for these reference compounds was found for tideglusib with a binding free energy value (ΔG) of −6.64 kcal/mol, while PX-12, disulfiram and shikonin were indicated in our calculations as possessing only weak binding affinities with a ΔG values above −4.0 kcal/mol. Interestingly, the experimental IC_50_ values obtained for these compounds also suggested them to be the least binding out of the eight experimentally validated 3CL^pro^ inhibitors [16].

In the sections that follow, we discuss the 3CL^pro^ substrate-site binding of the best predictions. In order to understand the 3CL^pro^–compound interactions, we plotted the protein–ligand interactions using LigPlot+ [36,37]. While all front runner compounds exhibit strong favorable interactions with the substrate binding site of 3CL^pro^, we have decided to accord special attention to those compounds that additionally interact with at least one of the two catalytic dyad residues, as well as to certain other compounds identified in the present work, such as steroid hormones that are of special interest in the ongoing global epidemic. The binding site because of its peculiar topology enables the binding of various chemical groups at different subsites within the rather large accommodating substrate site. We expect that the overall usefulness and predictive power of computational efforts should factor both the test compounds ability to bind strongly within the active site (indicated by the computed ΔG) as well as the ability to make specific contacts with amino acids within the active site, with special emphasis given to the catalytic dyad residues H41 and C145 (indicated by the computed distance to the 3CL^pro^ catalytic dyad, *d*_dyad_).

### 2.1. Screening of the Synthetic Compounds Library

We split the entire data set of 1,227,186 screened compounds into screening group A (sGrA) and screening group B (sGrB). The set sGrA is made up of 1,068,161 synthetic compounds that we first screened against the crystallographic structure of the 3CL^pro^ enzyme. Following this screening, we exerted a purely energetic cutoff (ΔG of −8.0 kcal/mol) to select the most promising ligands, yielding a total of 9515 synthetic compounds from the sGrA library. The choice of the cutoff was to improve the chances of attaining compounds with binding strengths significantly superior to those obtained for the reference inhibitors, for which the best binding was found for tideglusib with a ΔG of −6.64 kcal/mol.

We then analyzed the physicochemical properties of the 9515 virtual hits. They belong to several distinct chemical groups with molecular weights (MW) ranging from 200 to about 1000 g/mol. Since the intention at this time is to identify any compounds with the potential to bind to the active site of 3CL^pro^ regardless of their pharmacokinetic attributes, we have decided to deprioritize filtering based on physicochemical attributes usually employed in predicting compatibility with the oral route of administration. We analyzed the dominant ligand chemical fragments to find out if any of them featured more disproportionately than others. Figure S1 (Supplementary Materials) shows that the preponderant chemical fragments are mono- and bicyclic rings, many of them being heterocycles, which are chemical motifs generally associated with known drug molecules.

To account for the influence of protein flexibility in ligand binding, we performed a 100 ns MD simulation of the 3CL^pro^-N3 complex in water and clustered the active site conformations sampled during that simulation. The five most dominant conformations after removing the bound N3 were then employed for a rescreening of the 9515 compounds selected from the initial screening against the protein crystal structure. The ΔG values and distances between the bound ligand and the 3CL^pro^ catalytic dyad, *d*_dyad_, obtained for each compound were averaged over the five protease conformations. The resulting two values per compound were then employed in ranking the screened library and selecting the best compounds for further analysis. Introduction of receptor dynamics was observed to reduce the ability of many of the compounds to strongly interact with 3CL^pro^. While the docking against the crystal structure did not allow to discriminate between good and bad binders based on MW, after ensemble docking no compound with a MW higher than 600 g/mol returned computed affinities equal to or less than −7.0 kcal/mol. Moreover, only a few such high MW compounds were found to interact with the catalytic dyad within a 3.5 Å binding distance. On the other hand, compounds with small MW also turned out to be poor binders, dominating the compounds with binding free energy values above −5.0 kcal/mol.

**Figure 2:**
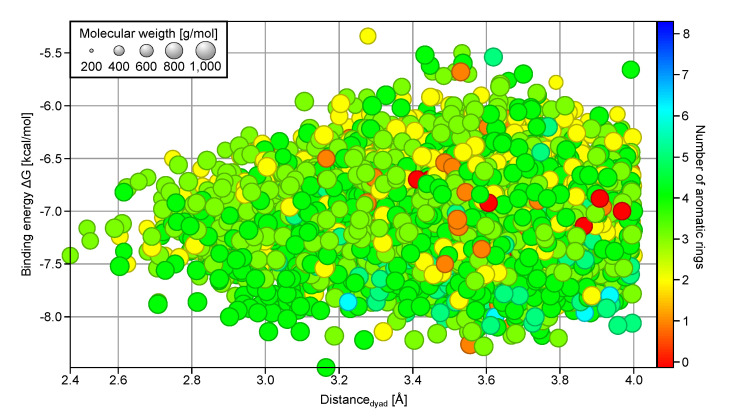
Distribution of the best binding synthetic compounds in terms of d_dyad_ (x-axis), ΔG (y-axis), number of aromatic rings (color), and molecular weight (circle size). The ΔG and *d*_dyad_ values are averages obtained from ensemble docking. Only compounds with ΔG<−5.0 kcal/mol and $d_{\mathrm{dyad}}\leq 4$ Å are shown.

A 4D plot showing the relationship between ΔG, *d*_dyad_, MW, and the number of aromatic rings present (Figure 2, only compounds with ΔG<−5.0 kcal/mol and $d_{\mathrm{dyad}}<4$ Å are shown) reveals that compounds with fewer than two aromatic rings (orange and red) are seldom found in the top binder subset (ΔG≤−7.5 kcal/mol); instead they are clustered around −6.5 kcal/mol. On the other hand, many of the compounds with more than four aromatic rings in their chemical structures (cyan and blue) have ΔG values below −7.5 kcal/mol. The number of aromatic rings also plays a role in determining the ability of a ligand to interact with H41 and C145 of the catalytic dyad, where fewer than five rings appear to be advantageous using $d_{\mathrm{dyad}}\leq 3.2$ Å as cutoff. Analysis of the structural fragments present in the good protease binders with ΔG≤−7.5 kcal/mol and $d_{\mathrm{dyad}}\leq 3.5$ Å (Figure S2) reveals an increased preference for bicyclic aromatic ring structures. Most of them are heterocycles with the majority having nitrogen atoms as heteroatoms, followed by oxygen atoms. This suggests a structural selection by the SARS-CoV-2 3CL^pro^ binding site for three or four aromatic, often hetero- and bicyclic rings and a MW between 400 and 600 g/mol.

**Figure 3:**
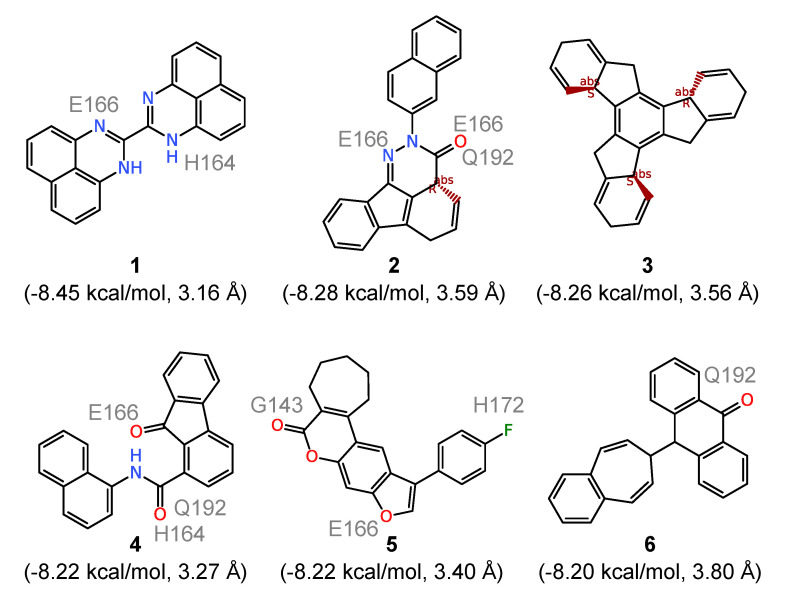
Synthetic compounds with the highest computed affinities for the substrate site of the SARS-CoV-2 3CL^pro^ enzyme obtained from ensemble docking. The average ΔG and $d_{\mathrm{dyad}}$ values are given in parentheses while ligand atoms involved in hydrogen bonding with 3CL^pro^ binding site residues (indicated) are also shown.

To further analyze the binding of the ligands to the substrate site of 3CL^pro^, we evaluated the six compounds with the strongest computed affinities (ΔG≤−8.2 kcal/mol) in detail. They are characterized by an essentially rigid core featuring polycyclic systems with three (compounds **1**, **4**, and **6** in Figure 3), four (compounds **2** and **5**), and seven (compound **3**) fused rings. The presence of these relatively large hydrophobic units encourages contacts with multiple amino acid residues within the 3CL^pro^ substrate site. However, none of them directly forms hydrogen bonds with the catalytic dyad residues and the respective distances are generally all above 3 Å. Counting the number of hydrogen bonds formed with the binding site residues, all six compounds form a total of sixteen such hydrogen bonds between them, involving G143, H164, E166, H170 and Q192. The very low ΔG values recorded for them appears to result majorly from hydrophobic interactions, especially for compound **3** which is a pure hydrocarbon (no functionalization) and thus should serve no more than as a useful probe for exploring the limits of shape and size compatible with the substrate site. Comparison with the electrostatic potential (ESP) of 3CL^pro^ in Figure 1B further supports the observation that for such compounds the binding is mainly driven by hydrophobic interactions since at the entry to the active site, where most of them are bound, the ESP is close to zero, whereas it is partly negative in the active site defined by the catalytic dyad.

**Figure 4:**
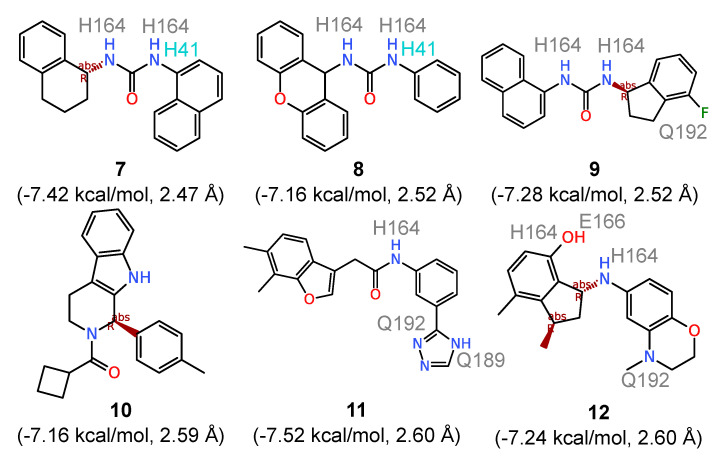
Synthetic compounds with the most intimate interaction with the catalytic dyad of the SARS-CoV-2 3CL^pro^ enzyme obtained from ensemble docking. The average ΔG and $d_{\mathrm{dyad}}$ values are given in parentheses while ligand atoms involved in hydrogen bonding with 3CL^pro^ binding site residues (indicated) are also shown.

To identify the compounds that most intimately interact with the catalytic dyad, all ligands with an average ΔG≤−7.0 kcal/mol were ranked based on distance from the catalytic dyad. The top six compounds that bind directly to the catalytic dyad (*d*_dyad_ ranging from 2.47 Å to 2.60 Å) are shown in Figure 4. Comparing these compounds with those with the highest calculated affinities, three structural patterns immediately emerge. First, the direct catalytic dyad binders feature smaller fused ring systems with a maximum of three fused rings (compounds **8** and **10** in Figure 4) and the majority having two fused rings (compounds **7**, **9**, **10**, and **12**). Second, all six compounds in Figure 4 have flexible substructures that allow them to adopt to the conformation needed to interact with the catalytic H41 and C145. In compounds **7**, **8**, and **9**, the superimposition of the three bound compounds (not shown) revealed a perfect alignment of their urea groups which make it possible to establish hydrogen-bonding contacts with the catalytic dyad. Third, five of the six ligands feature an amide group and all of them have at least one NH group, allowing for hydrogen bonding with the substrate site of 3CL^pro^. The cumulative number of hydrogen bonds established with the binding site residues across the five 3CL^pro^ conformations produced a total of thirty-three hydrogen bonding contacts shared between compounds **7** to **12**. This is more than twice the number recorded for compounds **1** to **6** with rigid structures. The amino acids involved in hydrogen bonding include H41 of the catalytic dyad, H164, E166, Q189 and Q192. Counting how frequently the two ligand groups make hydrophobic contacts with the two catalytic dyad residues indicates equal numbers of contacts with H41 (*N = 29* for both **1‒6** and **7‒12**), while compounds **7** to **12** demonstrate a higher contact frequency with C145 with a cumulative 27 contacts compared with 21 contacts obtained for compounds **1** to **6**. Thus, structural flexibility appears to improve both the hydrogen bonding interaction with the 3CL^pro^ binding site as well as hydrophobic contacts with the catalytic dyad residue C145.

The knowledge of the binding poses adopted by the rigid binders in Figure 3 and the flexible binders in Figure 4 along with the analysis of the physicochemical properties of good binders in Figure 2 and the dissection of their chemical fragments in Figure S2 can help facilitate the design of 3CL^pro^ specific inhibitors.

### 2.2. Interaction of Approved and Investigational Drugs with 3CL^pro^

**Figure 5:**
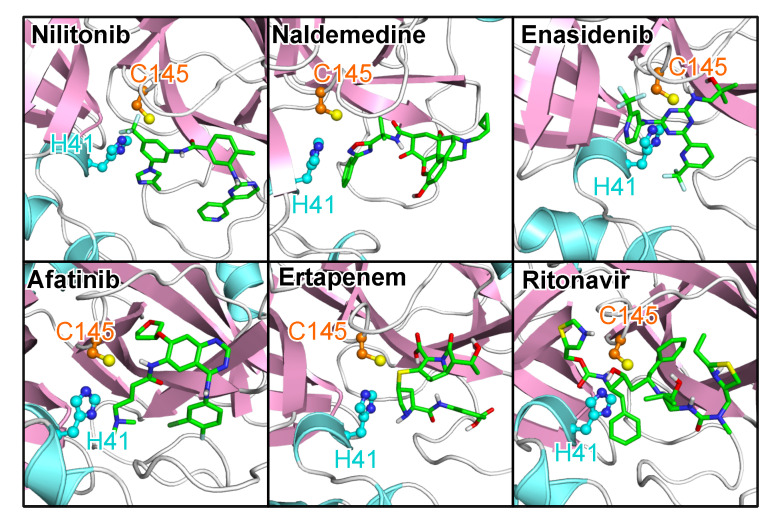
The poses of the five best Food and Drug Administration (FDA)-approved drugs and ritonavir. The top five compounds bind well to the substrate site in terms of both ΔG and closeness to the catalytic dyad of 3CL^pro^. The same protein and ligand representation as well as color scheme as in Figure 1 are used.

A total of 10,090 drugs were screened against both the crystallographic structure and the five MD-generated conformations of the SARS-CoV-2 3CL^pro^ enzyme. The screened compounds comprised drugs approved by the United States of America’s regulatory agency, FDA and those approved by other countries. Investigational drugs being trialed for various clinical indications were also screened. After the ensemble docking phase, we ranked the drug molecules based on their average ΔG and $d_{\mathrm{dyad}}$ values. The resulting top binding drug molecules are listed in Table S1, where FDA-approved and other drugs are separately listed. In the top ten based on cluster-averaged ΔG values there is only one FDA-approved drug, which is nilotinib (Table S1, entry 1), a tyrosine kinase inhibitor, with ΔG=−8.66 kcal/mol (Figure 5). Interestingly, the compound found to display the strongest 3CL^pro^ binding with ΔG=−10.46 kcal/mol is phthalocyanine (Table S1 entry 62), a non-toxic dye (IC_50_ of 10 g/kg) with biomedical applications including use as photosensitizer for non-invasive cancer therapy. The planar macrocyclic tetrapyrrole structure of phthalocyanine, coupled with the relatively extensive nature of the substrate binding site allows the therapeutic dye adopt two alternate binding poses (Figure S3). The first arrangement involves a purely hydrophobic contact with C145, with the ligand’s planar axis perpendicular to the H41–C145 axis. The alternate binding arrangement aligns the planar axis of the ligand parallel to the catalytic dyad axis. In this arrangement, the two polar hydrogen atoms at the center of the tetrapyrrole structure of phthalocyanine can form a hydrogen bond with H41. A similar binding pattern as for phthalocyanine was obtained for the second best binder, hypericin with ΔG=−9.12 kcal/mol (Table S1, entry 63). Hypericin also possesses a rigid and planar naphthodianthrone structure and in its 3CL^pro^ bound poses we observed it to alternate between a parallel arrangement enabling hydrophobic contact with the catalytic dyad and a perpendicular arrangement that makes hydrogen bond connection with H41 possible (Figure S3). It is of interest to point out that hypericin has been reported to possess a various spectrum of antiviral properties including inhibition of the replication of a coronavirus, the infectious bronchitis virus as reported in December 2019 by a Chinese group [38]. Islam et al. employed computational methods directed at forty known antiviral phytochemicals – hypericin is one of the principal active constituents of Saint John’s wort – and also identified hypericin as a good candidate for inhibiting the 3CL^pro^ enzyme of SARS-CoV-2 [39]. Other non-FDA drugs that outperform the best FDA-approved 3CL^pro^ ligand, that is, nilotinib in terms of ΔG include adozelesin (ΔG=−8.84 kcal/mol), telomestatin (ΔG=−8.80 kcal/mol), MK-3207 (ΔG=−8.74 kcal/mol), and radotinib (ΔG=−8.68 kcal/mol) (Table S1, entries 64–67).

**Figure 6:**
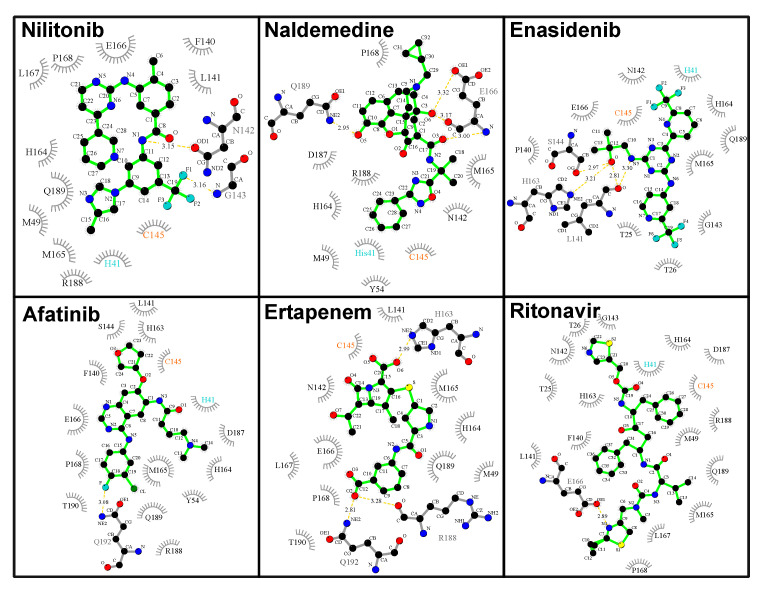
The 3CL^pro^–compound interactions for the five best FDA-approved drugs and ritonavir. The interactions were analyzed and plotted with LigPlot+ [36,37]. Hydrogen bonds are indicated by orange dashed lines between the atoms involved and the donor–acceptor distance is given in Å, while hydrophobic contacts are represented by gray arcs with spokes radiating towards the ligand atoms they contact. The contacted atoms are shown with spokes radiating back.

Of the top-performing FDA-approved drugs, four that directly interact with the 3CL^pro^ catalytic dyad are of special interest (Figure 5), and these include the opioid receptor antagonist naldemedine (ΔG=−8.06 kcal/mol, $d_{\mathrm{dyad}}=2.98$ Å), enasidenib indicated for the treatment of acute myeloid leukemia (ΔG=−7.76 kcal/mol, $d_{\mathrm{dyad}}=2.89$ Å), afatinib, an orally active tyrosine kinase inhibitor used in the treatment of non-small cell lung carcinoma (ΔG=−7.44 kcal/mol, $d_{\mathrm{dyad}}=3.04$ Å), and ertapenem, an antibiotic (ΔG=−7.24 kcal/mol, $d_{\mathrm{dyad}}=2.95$ Å). Apart from these four and nilotinib with the lowest energy value, ritonavir (ΔG=−6.70 kcal/mol, $d_{\mathrm{dyad}}=3.52$ Å), an antiviral agent currently being investigated for COVID-19 treatment [40], also desires special mention (Figure 5). Both nilotinib and afatinib are tyrosine kinase inhibitors, and interestingly this particular class of drugs has been shown to possess inhibitory activities against related coronaviruses [41]. Analysis of the interaction of the two tyrosine kinase inhibitors (Figure 6) shows effective catalytic dyad contacts between the trifluoromethyl group of nilotinib and C145, while its imidazole ring makes another contact with H41. While afatinib also makes contacts with both catalytic dyad residues, the fewer contacts it makes with other substrate site residues accounts for its slightly lower affinity. All the remaining four drugs presented in Figure 5 and Figure 6 make vital contacts with the two catalytic dyad residues with the exception of ertapenem which makes contact with only C145. Ritonavir as well as other agents like imatinib (entry 36 in Table S1) and sildernafil (entry 43 in Table S1) are currently going through clinical evaluation for the possible treatment of COVID-19 [42,43].

The screened non-FDA approved drugs produced four drug compounds directly interacting with the catalytic dyad and these include UK-432,097, a selective adenosine A_2A_ receptor agonist with urea linker approved for the treatment of chronic obstructive pulmonary disease; R-343, an investigational agent with tyrosine kinase inhibitory activities; and amrubicin used in the treatment of lung cancer. The binding poses of these drug compounds in the substrate site of 3CL^pro^ and their interactions are shown in Figure S4. This figure also includes remdesivir, which is a broad-spectrum antiviral medication and is currently being tested as a specific treatment for COVID-19 [40].

### 2.3. Interaction of Natural Products with 3CL^pro^

**Figure 7:**
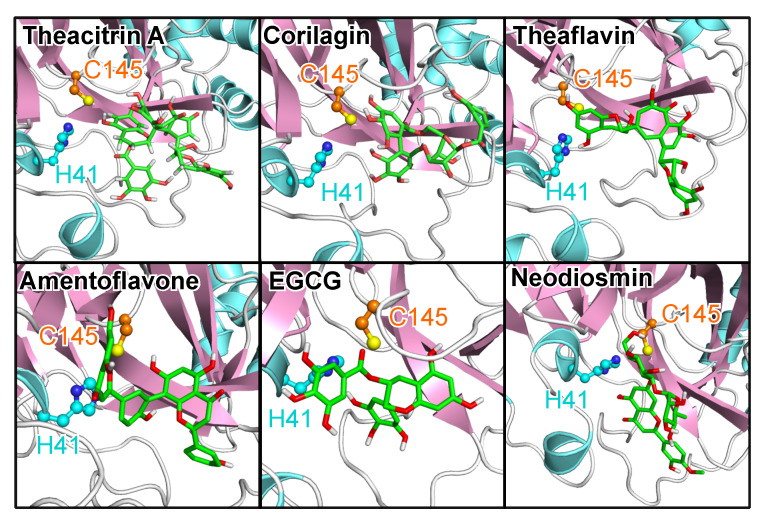
The poses of the six best natural drugs. The top six compounds bind well to the substrate site in terms of both ΔG and closeness to the catalytic dyad of 3CL^pro^. The same protein and ligand representation as well as color scheme as in Figure 1 are used.

The natural products that were screened were made up of the over 140,000 natural products retrieved from the ZINC library as well as over 3100 natural products of Nigerian plants origin. For consistent referencing we have quoted ZINC database [31,32,33] or PubChem (https://pubchem.ncbi.nlm.nih.gov/) references for all reported compounds. The natural products were screened against both the SARS-CoV-2 3CL^pro^ crystal structure and the MD-generated conformations of the enzyme. In Table S1 we have presented a total of 38 natural products demonstrating strong binding with both the crystal structure and the five ensemble structures. A significant percentage of the identified natural products are represented by flavonoids, some of which had previously been reported as possessing inhibitory activities against 3CL^pro^ of other coronavirus species. Figure 7 shows the binding poses of the top natural products in terms of both ΔG and *d*_dyad_, which are theacitrin A, corilagin, theaflavin, amentoflavone, epigallocatechin gallate (EGCG), and neodiosmin. It is pertinent to note that all six natural products, together with rhoifolin, daidzein, ginkgetin, proanthocyanidins and epitaraxerol, or their closely related derivatives and plant preparations have been reported to possess antiviral, and in some cases anti-coronavirus activities [44,45,46,47,48,49,50,51,52,53].

**Figure 8:**
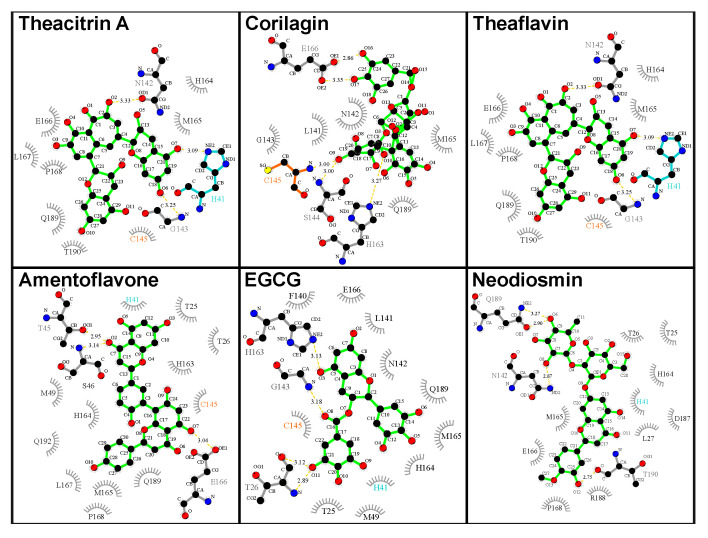
The 3CL^pro^–compound interactions for the six best natural drugs. The interactions were analyzed and plotted with LigPlot+ [36,37]. Hydrogen bonds are indicated by orange dashed lines between the atoms involved and the donor–acceptor distance is given in Å, while hydrophobic contacts are represented by gray arcs with spokes radiating towards the ligand atoms they contact. The contacted atoms are shown with spokes radiating back.

Analysis of the catalytic dyad interaction (Figure 8) revealed theacitrin A, theaflavin, and EGCG as being able to establish specific contacts with both H41 and C145, while corilagin only interacts with C145 and neodiosmin only with H41. Amentoflavone is the only one of the six selected compounds that did not form direct interactions with the catalytic dyad with respect to the 3CL^pro^ crystal structure (*d*_dyad_ > 6Å). This however changed when 3CL^pro^ dynamics was factored in, the binding distance averaged over five distinct 3CL^pro^ conformations diminished to 3.48 Å. In fact, after incorporating receptor flexibility none of the 38 top-performing natural products demonstrated catalytic dyad distances greater than 3.94 Å (Table S1).

Amentoflavone is a biflavonoid present in multiple plant sources such as *Ginkgo biloba* and St. John’s Wort; the latter one also harbors hypericin (see above). Amentoflavone represents one of the top-ranking candidates without an amide functional within its structure; in its place, it employs one of its phenolic groups in forming a hydrogen bond with T26, while establishing another hydrogen bond with H164. In addition, multiple hydrophobic contacts account for the strong binding to the 3CL^pro^ substrate site. Interestingly, amentoflavone, amidst its myriad pharmacological effects, was reported already in 2010 to possess micromolar range inhibitory activity against the main protease enzyme of SARS-CoV [50]. In the study, FRET analysis was employed in a bioactivity-guided isolation of four bioflavonoids and eight diterpenoids from the plant *Torreya nucifera*. Out of all isolated natural products, amentoflavone was found to demonstrate the most potent inhibition of the enzyme with an IC_50_ of 8.3 μM, while 280.8 μM inhibition was obtained for the monoflavonoid apigenin. This agreement with previously reported experimental observations specifically regarding the main protease target of a virus with a high similarity with SARS-CoV-2 provides an important validation for the computational results presented in this work. Amentoflavone has similarly been reported with antiviral activities mediated by interaction with other viral targets in viral infections including Dengue virus [54], Coxsackievirus B3 [55], the human immunodeficiency virus [56], and respiratory syncytial virus [57].

Glabrolide and zeylanone are other interesting natural products identified in our computational screening. Glabrolide (Table S1, entry 136) is found in herbs and spices such as *Glycyrrhiza glabra* (licorice) and *Glycyrrhiza uralensis* (Chinese licorice). While possessing structural similarity to the steroid structure, it differs from it by the replacement of the ring D with a six-membered ring and the possession of an extra lactone ring. In interacting with SARS-CoV-2 3CL^pro^, it establishes a hydrogen bond with T26 and several hydrophobic contacts, including a critical catalytic dyad contact with C145 (Figure S5). Zeylanone (Table S1, entry 117), on the other hand, employs its characteristic kink in its five-ring system to nestle comfortably within the hydrophobic cavity, forming a contact with C145, while at the same time establishing hydrogen bonds with the back bone atoms of F140 and E166 (Figure S5). Extracts of the originating plant, *Plumbago zeylanica*, were shown to possess inhibitory activities against Coxsackie virus B3, influenza A virus, and herpes simplex virus type 1 Kupka [58]. The pure zeylanone epoxide was reported to have inhibited influenza virus replication in vitro [59].

### 2.4. Interactions of Steroidal Hormones with 3CL^pro^

**Figure 9:**
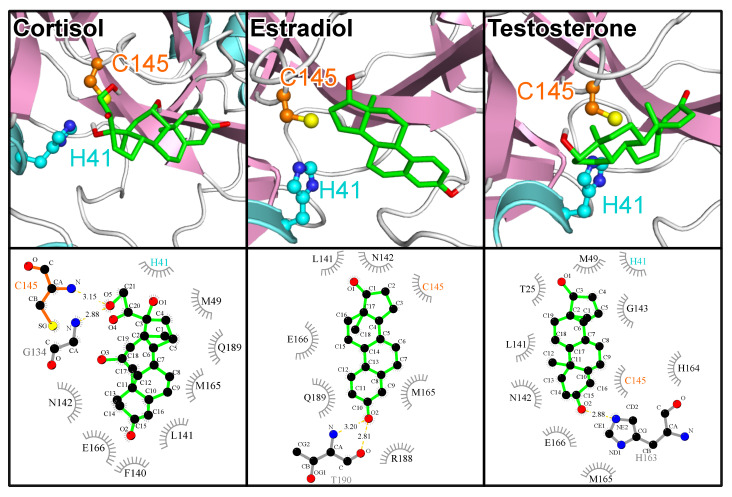
The poses and 3CL^pro^–compound interactions for three selected natural steroids. (**Top**) The binding poses of cortisol, estradiol, and testosterone are shown, using the same protein and ligand representations as well as color scheme as in Figure 1. (**Bottom**) The interactions were analyzed and plotted with LigPlot+ [36,37]. Hydrogen bonds are indicated by orange dashed lines between the atoms involved and the donor–acceptor distance is given in Å, while hydrophobic contacts are represented by gray arcs with spokes radiating towards the ligand atoms they contact. The contacted atoms are shown with spokes radiating back.

We profiled the interaction for the principal sex hormones to understand any possible modulatory role played by the steroids on 3CL^pro^-mediated SARS-CoV-2 viral replication. Not surprisingly given their high structural similarity, all investigated steroids demonstrated comparable binding affinities in interacting with the 3CL^pro^ enzyme (Table S1, entries 140–149). The endogenous steroidal hormones may thus act as natural inhibitors of 3CL^pro^. This includes cortisol, which was also among the best 3CL^pro^ binders in the FDA-approved drugs category. While estradiol with an averaged computed ΔG of −6.74 kcal/mol could be inferred as possessing a marginally superior binding strength compared to testosterone (ΔG=−6.52 kcal/mol), the observation that testosterone forms direct contacts with the two catalytic dyad residues (estradiol interacts with only C145) suggests that testosterone could have a stronger inhibitory effect on 3CL^pro^ (Figure 9).

However, it is unlikely that the 3CL^pro^ binding properties of the different sex hormones explain the gender-related differences that have been observed in vulnerability to COVID-19, but the possibility exists that the modulatory effects of sex hormones on viral proteins could be part of the complex phenomenon. This appears reasonable considering that age and gender-related differences characterizing human sex hormone levels have also been observed in susceptibility to the SARS-CoV-2 infectivity [60]. Other factors likely to contribute will include how other SARS-CoV-2 targets interact with the different hormones, the roles played by sex hormones in modulating immunological processes (especially testosterone), as well as the differential presence of structures like the testis that serves as additional reservoir for angiotensin-converting enzyme 2 (ACE2) needed for viral entry. It is possible that the presence of significantly higher titers of testosterone in younger men contribute to a relative inhibitory resistance against SARS-CoV-2 replication compared with elderly men with the characteristic functionally diminished testosterone levels, while the presence of additional ACE2-related SARS-CoV-2 entry sites in males’ testis perhaps explains the higher susceptibility relative to females. The male testis and adipose tissues represent sites in which ACE2 expression levels are reportedly highest in the human body [61]. In fact, the male testicles as well as obesity have been implicated in the higher vulnerability of male patients and obese individuals compared with female patients and non-obese people, respectively [62].

## 3. Conclusions

Using a computational drug design approach, we have identified a number of compounds whose ability to establish contacts with critical substrate site residues of 3CL^pro^ suggests them as good starting point for the design of inhibitors of SARS-CoV-2 replication. While most of the top-performing compounds were able to interact with impressive energetics, we have particularly focused on those compounds that form direct contacts with residues C145 and H41 that make up the catalytic dyad. The top-ranking compounds belong to diverse chemical classes and multiple structural features appear to underlie their ability to establish energetically favorable binding with the 3CL^pro^ substrate site. In order to gain a better understanding of the prerequisites for good 3CL^pro^ ligands, we dissected the top-ranked synthetic compounds with no current application as drugs into their fragments. This analysis revealed a clear preference for bicyclic, mostly aromatic rings with N and O atoms replacing some of the C atoms. Two or more of these rings are linked by flexible groups, which often bear NH groups and allow the ligands to optimally adopt to the geometry of the substrate site while hydrogen bonds can be formed via the various NH groups, N and O atoms. This knowledge can be used if one wishes to follow the rational drug design route for creating novel SARS-CoV-2 3CL^pro^ inhibitors.

A similar preference for multiple and multicyclic, mostly aromatic rings combined with flexible linker groups are also observed from our screening of existing drugs and natural products. Most of the best binding drugs are characterized by the presence of heterocycles where one or more C atoms are replaced with N (preferred) or O atoms. In addition, several of the rings bear functional groups such as carboxyl or trifluormethyl groups, while the linker groups often involve amide bonds. These groups allow the formation of hydrogen bonds with the amino acids in the substrate site of 3CL^pro^, while the amide bonds are peptidomimetics, resembling the natural substrate of 3CL^pro^. The best binding natural products are mostly bioflavonoids, which are also characterized by the presence of mono- and bicyclic aromatic rings which can change their orientation with respect to each other and thus adapt to the geometric requirements of the substrate binding site. However, unlike to the synthetic and drug compounds, the rings and functional groups of the bioflavonoids do not include nitrogen in any form, but a considerable amount of O atoms, either replacing C atoms in the ring structures or as part of functional hydroxyl groups. Especially, the latter lead to hydrogen bonds between the ligands and the substrate site of 3CL^pro^.

A similar finding as for the bioflavonoids was made for hypericin, which we identified as a good binding drug but does not involve any N atom. It is an anthraquinone derivative that is found in Saint John’s wort and is thus also a natural product. Hypericin is considered for the treatment of depressions and is believed to act as an antibiotic, antiviral and non-specific kinase inhibitor [63]. Interestingly, with amentoflavone we identified another natural product found in Saint John’s wort which may act as SARS-CoV-2 3CL^pro^ inhibitor. It should be noted that amentoflavone was reported a decade ago with micromolar range inhibitory effect on the closely related main protease enzyme of SARS-CoV [50]. This finding provides a validation for the virtual screening method incorporating protein dynamics employed in this work. Another interesting observation that we made is that several tyrosine kinase inhibitors were identified by our screening approach against 3CL^pro^. In addition to hypericin, this includes nilotinib, afatinib and R-343. Given that an earlier study reported another tyrosine kinase inhibitor, imatinib as inhibitor of SARS-CoV and MERS-CoV viral RNA expression [41], this class of inhibitors deserve further attention for finding a therapeutic for SARS-CoV-2. Finally, it should be mentioned that we identified many compounds that bind better to the main protease of SARS-CoV-2 than the eight reference compounds whose 3CL^pro^ inhibition capabilities were already demonstrated in vitro [16]. Ritonavir and remdesivir, which are currently tested as a treatment of COVID-19 [40], also bind to SARS-CoV-2. Interestingly, remdesivir which is thought to inhibit RNA-dependent RNA polymerase from SARS-CoV-2 binds better to the catalytic dyad of 3CL^pro^ than ritonavir, which is considered to be a protease inhibitor.

After we had identified cortisol among the drugs that exhibit good binding properties towards 3CL^pro^, we decided to screen all endogenous steoridal hormones and found that most of them bind well to the substrate site of 3CL^pro^. These hormones may thus act as natural inhibitors of the SARS-CoV-2 main protease, which would explain the age divide in terms of COVID-19 severity. This hypothesis is supported by the fact that progesterone was identified to be antiviral against SARS-CoV-2 [64]. Moreover, low testosterone levels in men are a risk factor for being more severely affected by COVID-19 than men with normal or high testosterone levels and women in general [60]. The question why postmenopausal women do not equally strongly suffer from COVID-19 might be answered by the different expression levels of ACE2, the receptor that SARS-CoV-2 uses for host entry and which in considerably amounts is expressed in testis [62]. A similar argument may account for the fact that COVID-19 is less prevalent in children [65], who have, like older people, low levels of sex hormones and may thus not rely on their possible protection from 3CL^pro^ inhibition. However, it was found that ACE2 is present in considerably lower quantities in children compared to adults above 18 years in age [66].

In the next state of our search for potent and clinically useful inhibitors of SARS-CoV-2 infection, we plan to employ further docking analysis involving other druggable COVID-19 targets, MD simulations to further assess the best docking predictions, as well as in vitro and cell-based assays to validate the outcomes of our the CADD data presented here.

## 4. Materials and Methods

We assembled a total of 1,227,186 ligand structural models principally from the ZINC database [31,32,33] which hosts 3D models of ligand molecules from other databases including the DrugBank library [34,35] as well as approved drugs (FDA and non-FDA), investigational drugs, and natural products including an in-house library of about 3200 natural products from Nigerian plants. This coverage is especially crucial for appropriately sampling the chemical scaffolds that are most fitting for interacting with the enzyme binding site. Other compounds, including the peptidomimetic inhibitor called N3, tideglusib, shikonin, cinanserin, ebselen, carmofur, disulfiram and PX-12 that had been reported to inhibit 3CL^pro^ of SARS-CoV-2 [16] were also included in our virtual screening runs as references. We next retrieved the X-ray crystallographic structure of the SARS-CoV-2 3CL^pro^ enzyme (PDB code 6LU7 [16]) from the RCSB website [67]. A docking grid allowing a 3.0 Å buffer region around the bound position of N3 in the 3CL^pro^ substrate site was generated with AutoDock Tool [68,69] and the compound library was subjected to virtual screening against the 3CL^pro^ crystal structure using AutoDock Vina [70] in multiple screening cycles.

In this first screening round, that we denote as screening group A (sGrA), 1,068,161 million compounds, mostly synthetic chemicals, were docked against the crystal structure. A maximum threshold of ΔG=−8.0 kcal/mol was applied to the resulting binding free energies, which yielded 9515 virtual hits that we selected to be part of the second screening group employing ensemble docking [71]. The second screening group B (sGrB) is composed of FDA-approved drugs (2099), other approved and investigational drugs (7991), natural products from the ZINC library (145,753), our in-house library of Nigerian phytochemicals (3182), and the best hits from sGrA (9515), leading to a total of 168,540 compounds. To account for protein flexibility in the second docking round, we performed a 100 ns MD simulation of the 3CL^pro^–N3 complex in solution using the MD software Gromacs 2018 [72] in combination with the AMBER14SB force field [73] and Parmbsc1 parameters [74] for modeling the protein, the generalized AMBER force field parameters (GAFF) [75] for N3, and the TIP3P water model [76] to explicitly simulate water. The resulting trajectory was subjected to geometric clustering [77] based on the 3CL^pro^ substrate binding site residues, which yielded 15 clusters. From those, the representative structures of the five most populated clusters (representing 88.1% of the dynamics) were employed for ensemble docking using the sGrB ligand screening group and the same docking settings as before.

**Figure 10:**
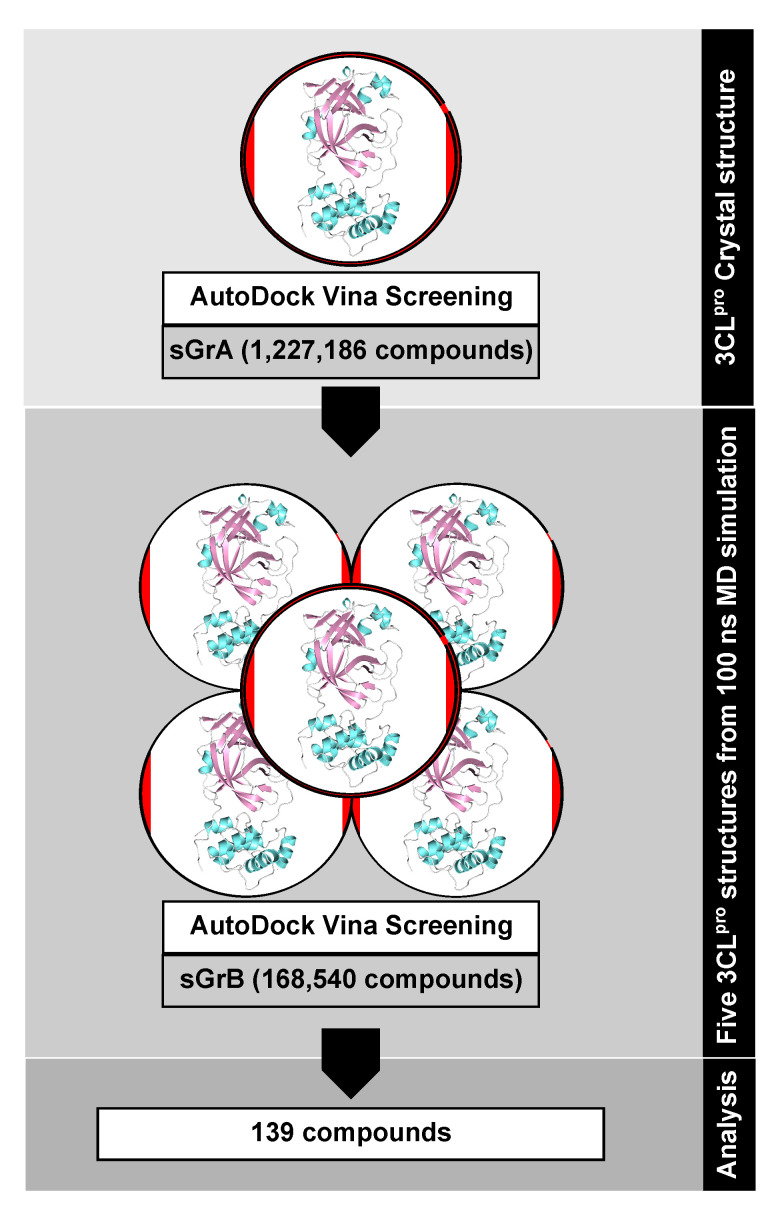
Flowchart of the virtual screening approach adopted in this work.

Figure 10 provides an overview of our virtual screening approach as a flowchart, while further technical details can be found in the Supplementary Materials. All 3D structures of the protein and ligands were created with PyMol [78], Figure 2, Figure 3 and Figure 4 were made with DataWarrior [79], and the protein‒ligand interactions were analyzed and plotted with LigPlot+ [36,37].

## Supplementary Materials

The following are available online at https://www.mdpi.com/1420-3049/25/14/3193/s1: Methods: Technical details for the docking and MD simulation. Figure S1: Chemical fragments majorly featured in the top performing 9515 synthetic compounds obtained from screening against the crystal structure of the SARS-CoV-2 main protease 3CL^pro^. Figure S2: Chemical fragments majorly featured in the top 2102 synthetic compounds obtained from ensemble docking and application of cutoff values of ΔG≤−7.0 kcal/mol and $d_{\mathrm{dyad}}\leq 3.5$ Å. Figure S3: The poses and 3CL^pro^–compound interactions of phthalocyanine and hypericin. Figure S4: The poses and 3CL^pro^–compound interactions of selected non-FDA-approved and investigational drugs. Figure S5: The poses and 3CL^pro^–compound interactions of zeylanone and glabrolide. Table S1: Names and properties of the compounds binding best to the active site of 3CL^pro^.
