# Supplementary material for: High Throughput Virtual Screening to Discover Inhibitors of the Main Protease of the Coronavirus SARS-CoV-2
Source: Molecules. 2020 Jul 13;25(14):3193. doi: 10.3390/molecules25143193 (PMC7396980; doi:10.3390/molecules25143193)
Supplement: Supplementary file 1 [file molecules-25-03193-s001.pdf]

Article

Supporting Materials

High throughput virtual screening to discover inhibitors of the main protease of the coronavirus SARS-CoV-2

Olujide O. Olubiyi [1,2,*], Maryam Olagunju [1], Monika Keutmann [1], Jennifer Loschwitz [1,3], and Birgit Strodel [1,3,*]

1 Institute of Biological Information Processing: Structural Biochemistry, Forschungszentrum Jülich, Jülich, Germany
2 Department of Pharmaceutical Chemistry, Faculty of Pharmacy, Obafemi Awolowo University, Ile-Ife, Nigeria
3 Institute of Theoretical and Computational Chemistry, Heinrich Heine University Düsseldorf, 40225 Düsseldorf, Germany
* Correspondence: olubiyioo@oauife.edu.ng, b.strodel@fz-juelich.de

Version 6th July 2020 submitted to Molecules

List of Figures

S1 Chemical fragments majorly featured in the top performing 9,515 synthetic compounds obtained from screening against the crystal structure of the SARS-CoV-2 main protease 3CL^{pro}. . . . 4
S2 Chemical fragments majorly featured in the top 2,102 synthetic compounds obtained from ensemble docking and application of cutoff values of $\Delta G \leq -7.0$ kcal/mol and $d_{dyad} \leq 3.5$ Å. . . . 4
S3 The poses and 3CL^{pro}–compound interactions of phthalocyanine and hypericin. . . . 5
S5 The poses and 3CL^{pro}–compound interactions of zeylanone and glabrolide. . . . 6
S4 The poses and 3CL^{pro}–compound interactions of selected non-FDA-approved and investigational drugs. . . . 7

List of Tables

S1 Names and properties of the compounds binding best to the active site of 3CL^{pro}. . . 8

Methods

Virtual screening protocol

We assembled a total of 1,227,186 ligand structural models principally from the ZINC database [1–3] which hosts 3D models of ligand molecules from other databases including the DrugBank library [4,5]. The screened ligand dataset includes existing drugs approved both by the United States Food and Drugs Administration (FDA) as well as by other countries regulatory authorities. In this work, we make clear distinction between FDA and non-FDA-approved drugs. Drugs still being investigated for various clinical indications represent another subset of our ligand library. A small but distinct subclass of our library consists of drugs being currently investigated for their activities (both validated and perceived) as anti-SARS-CoV-2 agents. More than 140,000 of the downloaded models are of natural products origin, while the largest part of the ZINC database-downloaded models contain synthetic chemical compounds. Apart from the ZINC database models, almost 3,200 models of natural products from Nigerian plants were obtained from an in-house database. While we did not deliberately characterize the screening virtual library based on chemical classes, we assumed that inclusion of compounds from multiple sources would achieve a decent coverage of sufficiently diverse chemical classes. This coverage is especially crucial for appropriately sampling the chemical scaffolds that are most fitting for interacting with the enzyme binding site. For the same reason and the need to look beyond the present state of understanding of the $3CL^{pro}$ enzyme, we have not selected a screening protocol focused solely on known protease inhibitors. Other compounds, including the peptidomimetic inhibitor called N3, tideglusib, shikonin, cinanserin, ebselen, carmofur, disulfiram and PX-12 that had been reported to inhibit $3CL^{pro}$ of SARS-CoV-2 [6] were also included in our virtual screening runs as references. Random visual inspection of the ligand models obtained from the different sources indicated *prima facie* structural correctness, and the isolated instances of inappropriate model (e.g., replacing esterified candesartan currently in clinical trials with candesartan) were treated on individual bases.

We retrieved the X-ray crystallographic structure of the SARS-CoV-2 ($3CL^{pro}$) enzyme (PDB code 6LU7 [6]) from the RCSB website [7]. The structure represents the functional form of $3CL^{pro}$ in complex with the inhibitor N3 at a 2.16 Å resolution. In the structure, the peptidomimetic inhibitor could be seen making contacts with critical substrate binding site residues including the C145–H41 catalytic dyad. A docking grid allowing a 3.0 Å buffer region around the bound position of N3 in the $3CL^{pro}$ substrate site was generated with AutoDock [8,9] Tool and centered at x, y, z-position of -10.745 Å, 12.33 Å, 68.84 Å. The buffer region serves to accommodate different molecular sizes covered in the screening library. After adding hydrogen atoms to $3CL^{pro}$, Gasteiger partial charges were computed and added to $3CL^{pro}$ using AutoDock Tool [8,9] protein model was saved in the PDBQT format. PDBQT files were also generated for each of the ligands in the screening library, following which the library was subjected to virtual screening against the $3CL^{pro}$ molecule using AutoDock Vina [10], which treats the ligand as fully flexible while keeping the receptor rigid. Different cycles of screening were performed in a way that balances the complexity of the ligand libraries with computational resources on one hand, with the need to assure reliability of our computational model for the biochemical process (i.e., enzyme inhibition) being investigated. In the initial round, the screening group A (sGrA) composed of 1,068,161 million synthetic compounds from the ZINC database was screened against the $3CL^{pro}$ crystal structure. From sGrA, 9,515 virtual hits with computed binding free energy ΔG values less than or equal to -8.0 kcal/mol were selected for rescreening using ensemble docking [11]. The second screening group B (sGrB) that is composed of FDA-approved drugs (2,099) and other approved and investigational drugs (7,991), as well as natural products from ZINC library (145,753) and from our in-house library of secondary metabolites from Nigerian plants (3,182), and reference compounds was similarly screened against the $3CL^{pro}$ crystal structure and as complete set submitted to the second round of screening with ensemble docking.

Virtual rescreening protocol – incorporating protein dynamics.

To account for protein flexibility in the second docking round, we performed ensemble docking [11] on both the hit list generated from sGrA ($N = 9,515$) and the entire sGrB containing a total of 159,025 compounds. To this end, we performed a 100 ns MD simulation of the 3CL^{pro}–N3 complex in solution using the MD software Gromacs 2018 [12]. As protein force field AMBER14SB [13] with Parmbsc1 parameters [14] was used and combined with the TIP3P water model [15] to explicitly simulate water. For N3, the generalized AMBER force field parameters (GAFF) [16] were used. To derive the parameters, we conducted quantum mechanics optimization at the HF6-31G* level of theory with Gaussian 09 [17] and used the restrained electrostatic potential (RESP) method to calculate partial charges [18,19] via Antechamber[20] available in AmberTool 19 [21]. To generate the GROMACS input files for N3, we used ACPYPE tool [22]. As starting structure, the 3CL^{pro}–N3 crystal structure was taken (PDB code 6LU7) [6], which was centered in a cubic box of size 80 × 80 × 80 Å^3, solvated, and NaCl was added at a concentration of 150 mM while at the same time neutralizing the system, resulting in a system size of 50,966 atoms in total. The system was first energy-minimized using a steepest descent algorithm, equilibrated in the *NVT* ensemble (i.e., with a constant number of molecules, volume, and temperature) for 0.1 ns and then for 1 ns in the *NpT* ensemble at $T = 310$ K (Nosé-Hoover thermostat [23,24]) and $p = 1.0$ bar (Parrinello-Rahman barostat [25]). The production MD simulation was performed for 100 ns with the same parameters as in the *NpT* equilibration. Electrostatic interactions were treated with the particle-mesh Ewald method [26,27] in conjunction with periodic boundary conditions and a real-space cutoff of 12 Å. The Lennard-Jones interactions were also cut at 12 Å. A leapfrog stochastic dynamics integrator was used for the integration of the equations of motion using a time step of 2 fs. The LINCS algorithm [28] was used to constrain all bond lengths during the MD simulation. The resulting trajectory was subjected to geometric clustering using the Daura algorithm [29] based on the conformations sampled by 3CL^{pro} substrate binding site residues (within 10 Å of N3). With a clustering cutoff of 1.5 Å, 15 clusters were identified, from which the representative structures of the five most populated clusters representing 88.1% of the dynamics were selected for ensemble docking. As for the docking against the 3CL^{pro} crystal structure, a docking grid was generated for each of the five MD-generated 3CL^{pro} conformations with a 3.0 Å buffer around the bound position of N3. The selected hits from the sGrA as well as the entire sGrB group were then subjected to another round of screening against the five MD-generated 3CL^{pro} conformers using AutoDock Vina. The results were subsequently analyzed to understand critical structural and physicochemical aspects of 3CL^{pro} enzyme inhibition that may benefit rational design of inhibitors.

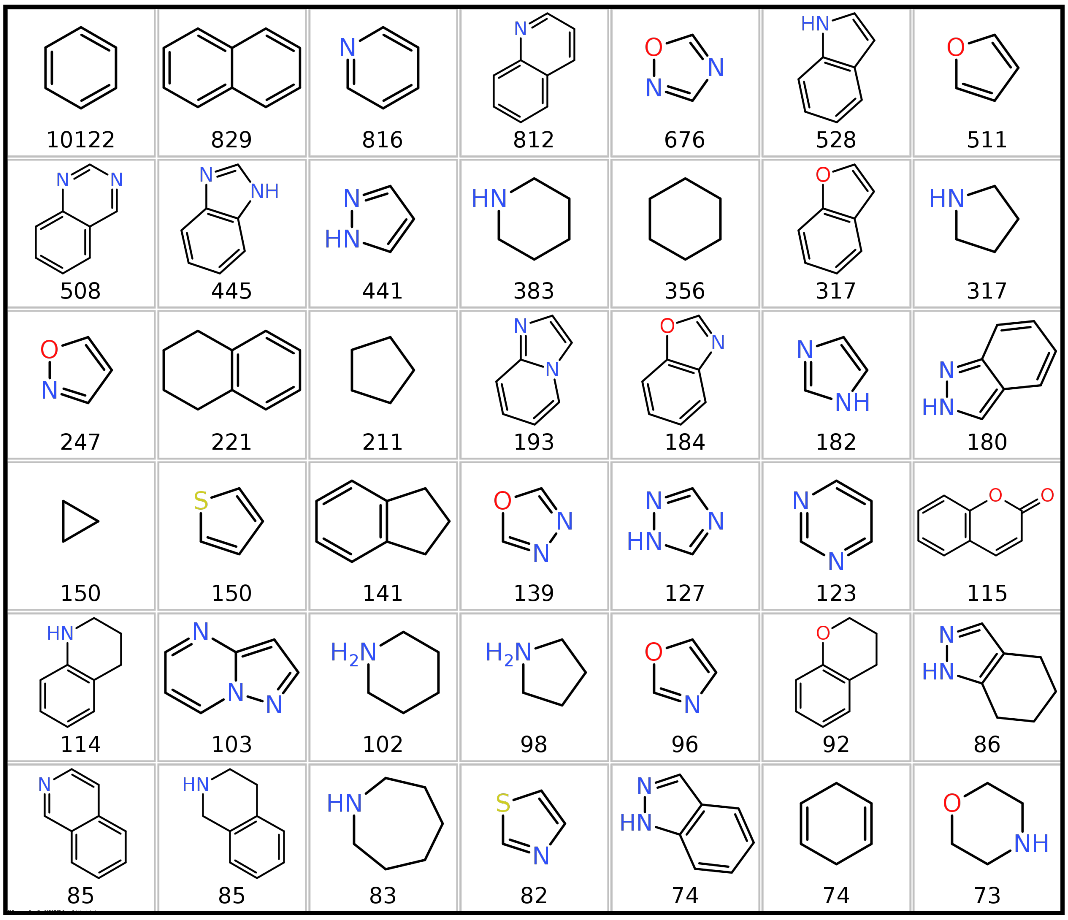

Figure S1. Chemical fragments majorly featured in the top performing 9,515 synthetic compounds obtained from screening against the crystal structure of the SARS-CoV-2 main protease 3CL^{pro}. The numbers represent the occurrence in absolute numbers.

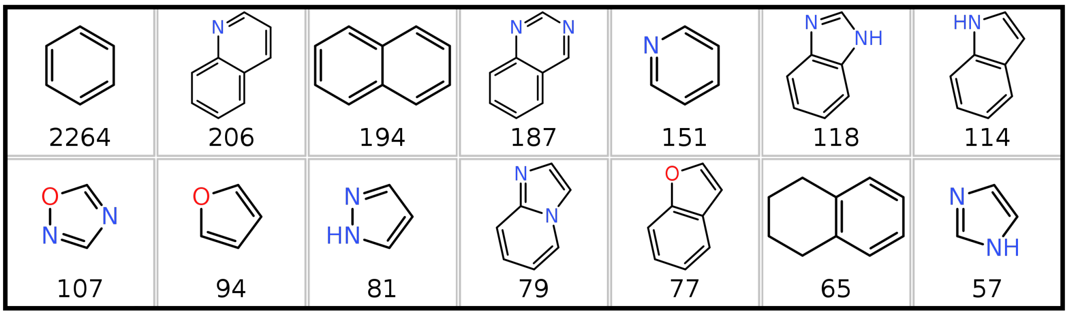

Figure S2. Chemical fragments majorly featured in the top 2,102 synthetic compounds obtained from ensemble docking and application of cutoff values of $\Delta G \leq -7.0$ kcal/mol and $d_{dyad} \leq 3.5$ Å. The numbers represent the occurrence in absolute numbers.

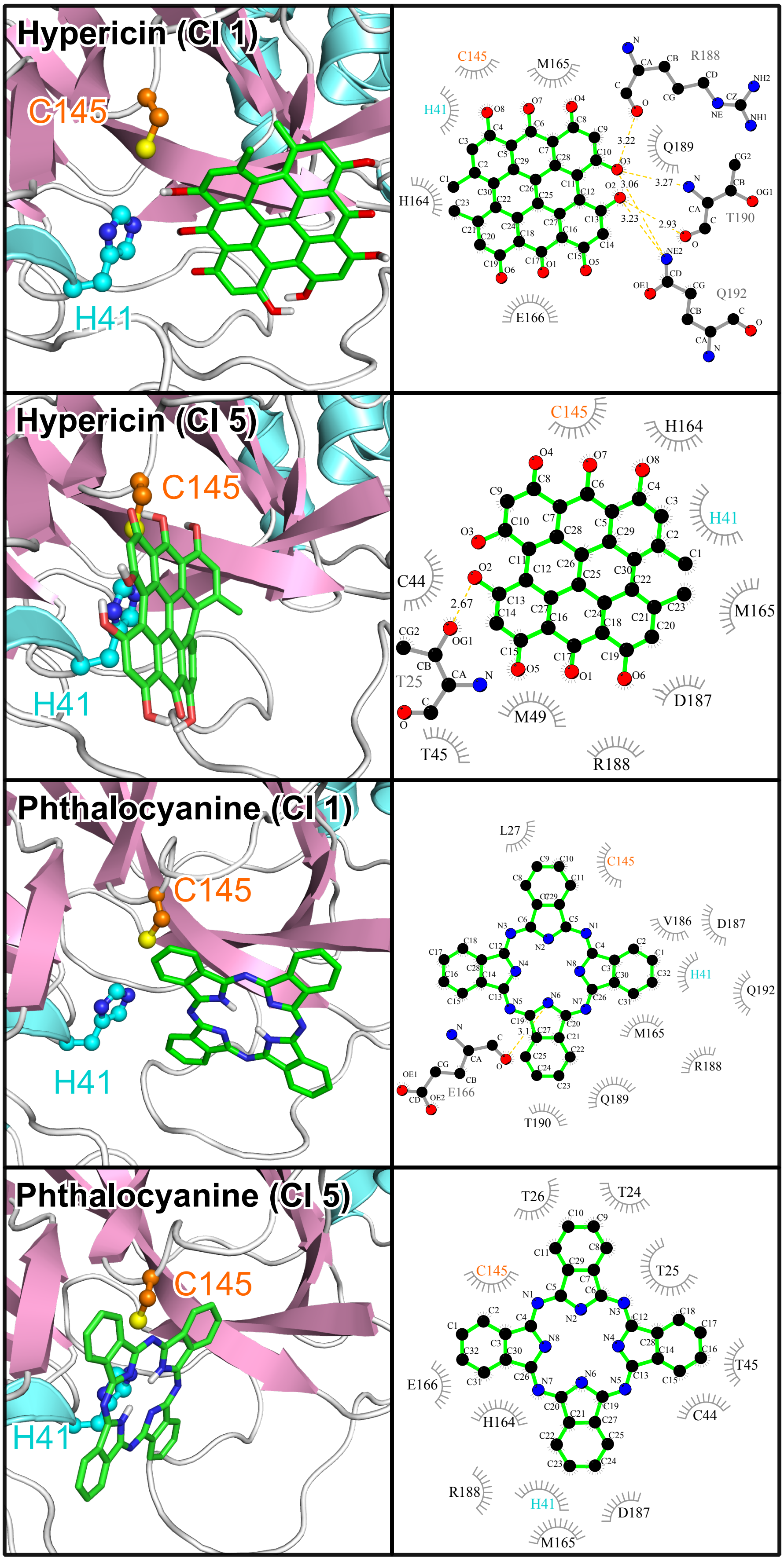

Figure S3. The poses and 3CL^{pro}–compound interactions of phthalocyanine and hypericin.

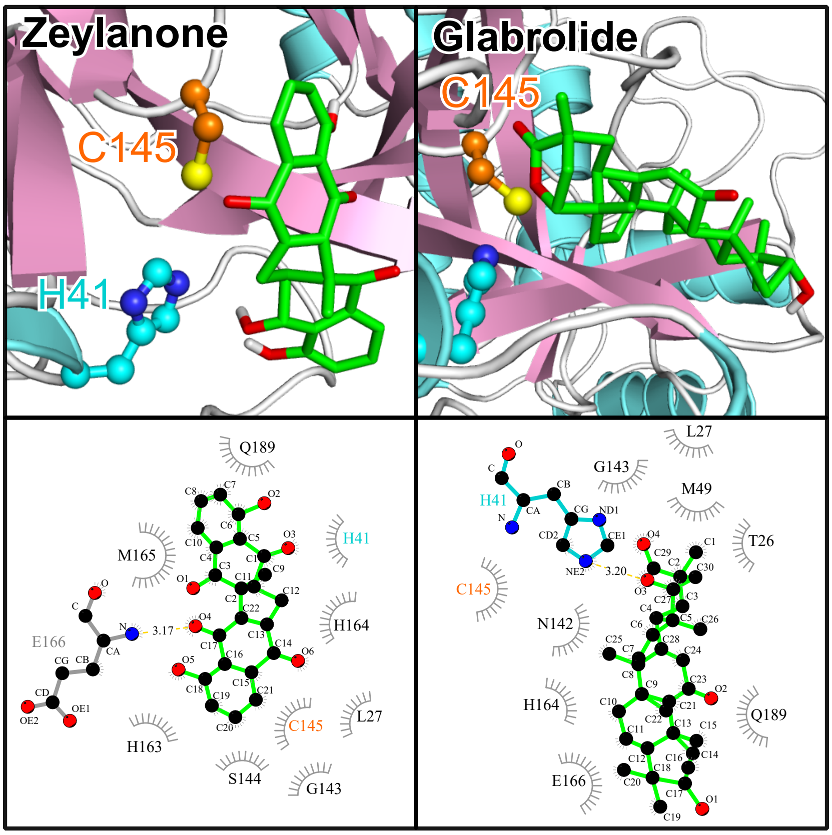

Figure S5. The poses and 3CL^{pro}–compound interactions of zeylanone and glabrolide.

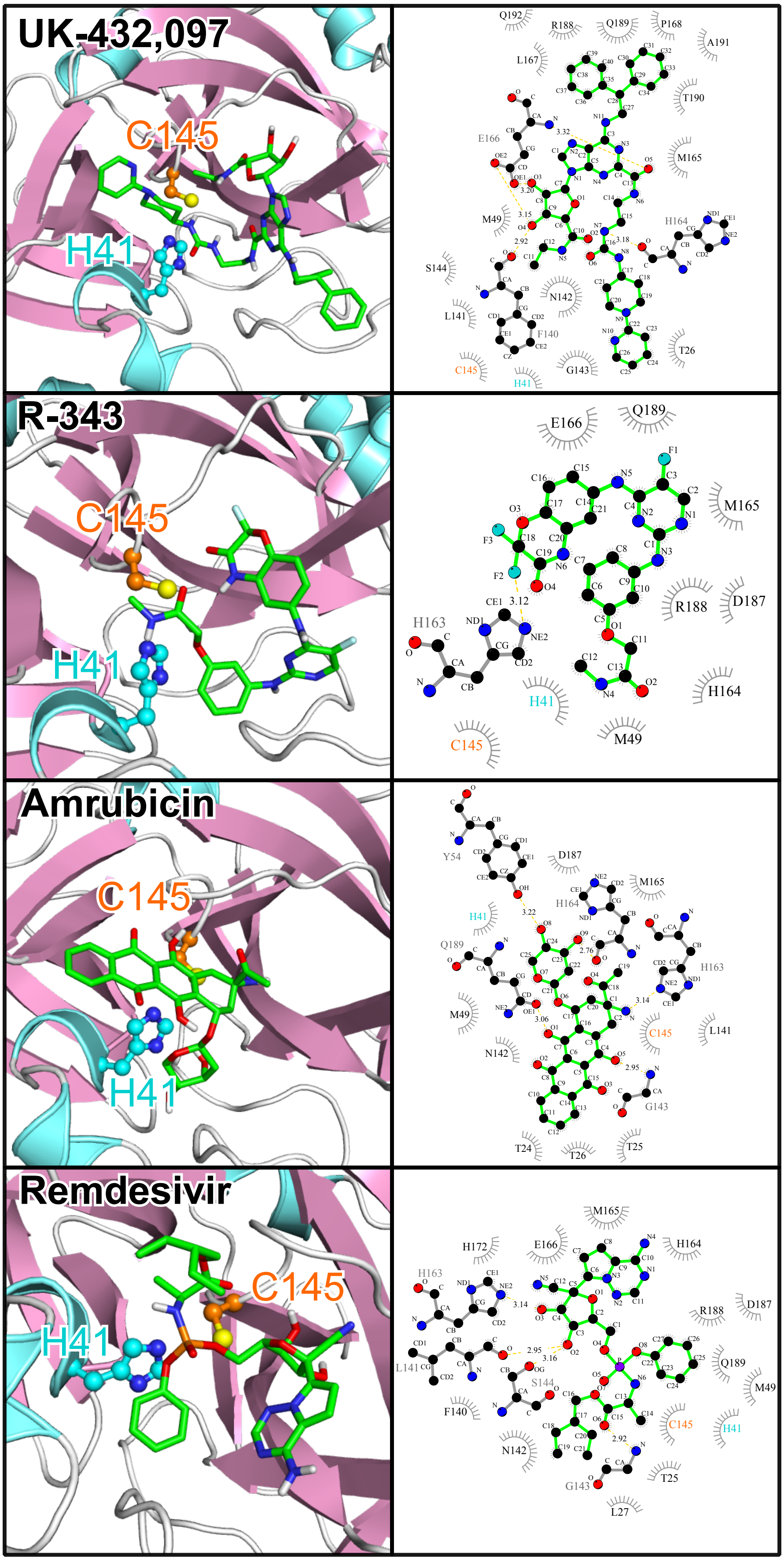

Figure S4. The poses and $3CL^{pro}$–compound interactions of selected non-FDA-approved and investigational drugs.

Table S1. Names and properties of the compounds binding best to the active site of 3CL^{pro}. These compounds include FDA-approved drugs, other drugs, natural products, steroids, and eight reference compounds. The ΔG values and distances to the catalytic dyad (d_{dyad}) are average values obtained from ensemble docking agains five representative structures of 3CL^{pro} obtained from MD simulation. The compounds highlighted in bold are discussed in more detail in the text.

No.	Accession ID	Compound name	ΔG [kcal/mol]	d_{dyad} [Å]
		**FDA drugs**		
**1**	**ZINC000006716957**	**Nilotinib**	**−8.66**	**3.43**
2	ZINC000064033452	Lumacaftor	−8.36	3.36
3	ZINC000018324776	Dutasteride	−8.36	3.65
4	ZINC000003993855	Tadalafil	−8.28	3.42
5	ZINC000052955754	Ergotamine	−8.10	3.72
**6**	**ZINC000100378061**	**Naldemedine**	**−8.06**	**2.98**
7	ZINC000003920266	Idarubicin	−8.04	3.25
8	ZINC000001530788	Simeprevir	−8.02	3.68
9	ZINC000013831130	Raltegravir	−7.98	3.35
10	ZINC000014210642	Azilsartan	−7.90	3.27
11	ZINC000058581064	Dolutegravir	−7.88	3.73
12	ZINC000003927822	Lurasidone	−7.80	3.53
13	ZINC000004214700	Paliperidone	−7.78	3.58
**14**	**ZINC000222731806**	**Enasidenib**	**−7.76**	**2.89**
15	ZINC000012503187	Conivaptan	−7.76	3.33
16	ZINC000001530886	Telmisartan	−7.74	3.35
17	ZINC000003938684	Etoposide	−7.72	3.53
18	ZINC000029416466	Saquinavir	−7.70	3.32
19	ZINC000072318121	Abemaciclib	−7.70	3.65
20	ZINC000008101127	Indocyanine	−7.68	3.52
21	ZINC000011617039	Pazopanib	−7.66	3.58
22	ZINC000003938686	Palbociclib	−7.66	3.61
23	ZINC000004099008	Teniposide	−7.66	3.67
24	ZINC000035328014	Ibrutinib	−7.64	3.45
25	ZINC000253632968	Cromolyn	−7.64	3.68
26	ZINC000043100709	Trematinib	−7.60	3.42
27	ZINC000003831151	Montelukast	−7.58	3.41
28	ZINC000022448696	Indinavir	−7.54	3.29
29	ZINC000040430143	Olaparib	−7.52	3.86
30	ZINC000049036447	Suvorexant	−7.50	3.53
**31**	**ZINC000003976838**	**Afatinib**	**−7.44**	**3.04**
32	ZINC000003816514	Rolapitant	−7.44	3.40
33	ZINC000013986658	Idelalisib	−7.42	3.26
34	ZINC000013818943	Regadenoson	−7.38	3.20
35	ZINC000100003902	Maraviroc	−7.38	3.59
36	ZINC000019632618	Imatinib	−7.36	3.11
37	ZINC000003827556	Delafloxacin	−7.34	3.14
38	ZINC000003986735	Dasatinib	−7.32	3.46
39	ZINC000027990463	Lomitapide	−7.32	3.79
40	ZINC000003932831	Candesartan	−7.32	3.64
41	ZINC000035902489	Crizotinib	−7.30	3.57
42	ZINC000004175630	Pimozide	−7.30	3.73
43	ZINC000019796168	Sildenafil	−7.28	3.55

44	ZINC000043206370	Niraparib	−7.26	3.46
**45**	**ZINC000003918453**	**Ertapenem**	**−7.24**	**2.95**
46	ZINC000003860453	Fluorescein	−7.22	3.56
47	ZINC000001481815	Deferasirox	−7.22	3.59
48	ZINC000018324776	Vardenafil	−7.20	3.62
49	ZINC000060325170	Cobimetinib	−7.18	3.16
50	ZINC000000537791	Glimepiride	−7.18	3.51
51	ZINC000001489478	Sitagliptin	−7.18	3.60
52	ZINC000003812865	Olsalazine	−7.18	3.67
53	ZINC000011677837	Apixaban	−7.12	3.78
54	ZINC000005844788	Nebivolol	−7.04	3.43
55	ZINC000000897240	Azelastine	−7.04	3.45
56	ZINC000100022637	Tipranavir	−6.92	3.37
57	ZINC000001552174	Cilostazol	−6.86	3.45
58	ZINC000030691797	Perampanel	−6.82	3.67
59	ZINC000085537017	Cangrelor	−6.44	3.28
**60**	**ZINC000003944422**	**Ritonavir**	**−6.70**	**3.52**
61	ZINC000001530948	Thalidomide	−6.26	3.83
		**Non-FDA and investigational drugs**		
**62**	**ZINC000012358610**	**Phthalocyanine**	**−10.46**	**3.63**
**63**	**ZINC000003780340**	**Hypericin**	**−9.12**	**2.85**
64	ZINC000003922429	Adozelesin	−8.84	3.84
65	ZINC000003975327	Telomestatin	−8.80	3.34
66	ZINC000043203371	MK-3207	−8.74	3.54
67	ZINC000059749972	Radotinib	−8.68	3.43
68	ZINC000003812168	Ruboxistaurin	−8.56	3.55
69	ZINC000003950115	TMC647055	−8.50	3.77
70	ZINC000095092808	—	−8.48	3.86
71	ZINC000049888572	—	−8.42	3.55
**72**	**ZINC000095539256**	**UK-432,097**	**−8.34**	**3.18**
**73**	**ZINC000038576002**	**R-343**	**−8.30**	**2.71**
74	ZINC000014880002	Dihydroergotoxine	−8.30	3.43
75	ZINC000004215648	Dihydroergocornine	−8.30	3.80
76	ZINC000003817327	Ly2090314	−8.30	3.83
77	ZINC000003781738	Lestaurtinib	−8.26	3.53
78	ZINC000254071113	Ciluprevir	−8.26	3.64
79	ZINC000063933734	Rebastinib	−8.24	3.36
80	ZINC000059185874	GDC-0834	−8.24	3.58
81	ZINC000043133316	Tirilazad	−8.24	3.87
82	ZINC000098208742	Entospletinib	−8.20	3.57
83	ZINC000018710085	—	−8.20	3.97
84	ZINC000003930598	—	−8.18	3.71
85	ZINC000004215770	Elsamitrucin	−8.12	3.31
**86**	**ZINC000003780800**	**Amrubicin**	**−8.10**	**2.96**
87	ZINC000001539348	—	−8.10	3.39
88	ZINC000003978083	Tubocurarine	−8.10	3.56
89	ZINC000068250462	Tucatinib	−8.10	3.88
90	ZINC000001494900	Enzastaurin	−8.08	3.48
91	ZINC000003950115	Lonafarnib	−8.08	3.73

92	ZINC000019899628	Fenoverine	−8.06	4.06
93	ZINC000095535868	Rwj-58259	−8.04	3.55
94	ZINC000001490807	—	−8.04	3.60
95	ZINC000006717782	BMS-599626	−8.04	3.66
96	ZINC000100001820	PF-00477736	−8.02	3.49
97	ZINC000028827350	Telcagepant	−8.20	3.58
98	ZINC000003973984	Sotrastaurin	−8.02	3.68
99	ZINC000021290045	—	−8.00	3.32
100	ZINC000100029945	Zosuquidar	−8.00	3.45
101	CID121304016	Remdesivir	−6.44	2.84
**Natural products from ZINC database and flavonoids**				
**102**	**ZINC000150352420**	**Theacitrin A**	**−9.82**	**3.31**
**103**	**ZINC000004098612**	**Corilagin**	**−9.58**	**3.22**
104	ZINC000008214976	Theasinensin B	−9.18	3.41
105	ZINC000169372863	Theasinensin A	−9.16	3.42
**106**	**ZINC000003978446**	**Theaflavin**	**−9.16**	**3.75**
107	ZINC000004235306	—	−9.18	3.52
108	ZINC000230071666	Theacitrin C	−8.96	3.54
**109**	**ZINC000003984030**	**Amentoflavone**	**−8.88**	**3.48**
110	ZINC000169333962	Theasinensin F	−8.78	3.43
111	ZINC000001531664	Ginkgetin	−8.76	3.68
112	ZINC000044351169	Proanthocyanidin A1	−8.76	3.74
113	ZINC000003978800	Rhoifolin	−8.70	3.56
114	ZINC000004098619	Proanthocyanidin A2	−8.68	3.69
115	ZINC000095619717	Proanthocyanidin A5′	−8.64	3.54
116	ZINC000003197535	Isoginkgetin	−8.54	3.67
**117**	**ZINC000014887561**	**Zeylanone**	**−8.44**	**3.56**
118	CID10077799	Isocorilagin	−8.28	3.81
**119**	**ZINC000003870412**	**Epigallocatechin gallate**	**−8.28**	**3.50**
120	ZINC000006624329	—	−8.28	3.77
121	ZINC000002148919	—	−8.26	3.63
122	ZINC000002107922	—	−8.24	3.82
123	ZINC000002161217	—	−8.22	3.48
124	ZINC000008297065	—	−8.20	3.67
125	ZINC000002125422	—	−8.18	3.50
126	ZINC000008764269	—	−8.16	3.50
127	ZINC000008789992	—	−8.12	3.56
128	ZINC000012296408	—	−8.10	3.59
129	ZINC000002147804	—	−8.08	3.55
130	ZINC000012881832	—	−8.06	3.46
131	ZINC000002158857	—	−8.06	3.46
132	CID5321811	Bavacoumestan A	−8.04	3.60
**133**	**ZINC000100828606**	**Neodiosmin**	**−8.04**	**3.12**
134	ZINC000011865175	—	−8.00	3.67
135	ZINC000002114470	—	−8.00	3.60
**136**	**ZINC000100777667**	**Glabrolide**	**−7.89**	**3.94**
137	CID12443227	Epitaraxerol	−7.00	3.79
138	ZINC000004098322	Homoeriodictyol	−6.64	3.42
139	ZINC000018847034	Daidzein	−6.04	3.75

		**Steroids**		
140	CID27125	Estetrol	−6.86	3.39
**141**	**ZINC000004340309**	**Cortisol**	**−6.80**	**3.62**
**142**	**CID5757**	**Estradiol**	**−6.74**	**3.41**
143	CID5994	Progesterone	−6.70	3.93
144	ZINC000004428526	Androstenedione	−6.66	3.65
145	CID91451	17-α-hydroxypregnanolone	−6.62	3.41
146	ZINC000003815419	2-Hydroxyestradiol	−6.46	3.67
147	ZINC000003807917	Dehydroepiandrosterone	−6.40	3.81
148	ZINC000004081043	Allopregnanolone	−6.34	3.88
149	**ZINC000118912393**	**Testosterone**	**−6.32**	**3.90**
		**Reference compounds**		
150	ZINC000013985228	Tideglusib	−6.64	3.71
151	PDB 6LU7	N3	−6.00	3.23
152	ZINC000001714738	Cinanserin	−5.90	3.54
153	CID3194	Ebselen	−5.74	4.17
154	ZINC000001542916	Carmofur	−5.60	3.64
155	ZINC000013209429	PX-12	−3.84	3.97
156	ZINC000001529266	Disulfiram	−3.80	3.45
157	ZINC000002015152	Shikonin	−2.72	3.59

References

1. Irwin, J.; Shoichet, B. ZINC–A Free Database of Commercially Available Compounds for Virtual Screening. *Journal of Chemical Information and Modeling* **2005**, *45*, 177–182.
2. Irwin, J.; Sterling, T.; Mysinger, M.; Bolstad, E.; Coleman, R. ZINC: A Free Tool to Discover Chemistry for Biology. *J. Chem. Inf. Model.* **2012**, *52*, 1757–1768.
3. Sterling, T.; Irwin, J. ZINC 15 – Ligand Discovery for Everyone. *J. Chem. Inf. Model.* **2015**, *55*, 2324–2337.
4. Wishart, D.; Knox, C.; Guo, A.; Shrivastava, S.; Hassanali, M.; Stothard, P.; Chang, Z.; Woolsey, J. DrugBank: a comprehensive resource for in silico drug discovery and exploration. *Nucleic Acids Res.*, *34*.
5. Wishart, D.; Feunang, Y.; Guo, A.; Lo, E.; Marcu, A.; Grant, J.; Sajed, T.; Johnson, D.; Li, C.; Sayeeda, Z.; Assempour, N.; Iynkkaran, I.; Liu, Y.; Maciejewski, A.; Gale, N.; Wilson, A.and Chin, L.; Cummings, R.; Le, D.; Pon, A.; Knox, C.; Wilson, M. DrugBank 5.0: a major update to the DrugBank database for 2018. *Nucleic Acids Res.* **2017**, *46*, D1074–D1082.
6. Jin, Z.; Du, X.; Xu, Y.; Deng, Y.; Liu, M.; Zhao, Y.; Zhang, B.; Li, X.; Zhang, L.; Peng, C.; Duan, Y.; Yu, J.; Wang, L.; Yang, K.; Liu, F.; Jiang, R.; Yang, X.; You, T.; Liu, X.; Yang, X.; Bai, F.; Liu, H.; Liu, X.; Guddat, L.W.; Xu, W.; Xiao, G.; Qin, C.; Shi, Z.; Jiang, H.; Rao, Z.; Yang, H. Structure of M^{pro} from COVID-19 virus and discovery of its inhibitors. *Nature* **2020**.
7. Berman, H.; Westbrook, J.; Feng, Z.; Gilliland, G.; Bhat, T.; Weissig, H.; Shindyalov, I.; Bourne, P. The Protein Data Bank. *Nucleic Acids Res.* **2000**, *28*, 235–242.
8. Goodsell, D.; Morris, G.; Olson, A. Automated docking of flexible ligands: Applications of AutoDock. *J. Mol. Recognit.* **1996**, *9*, 1–5.
9. Santos-Martins, D.; Forli, S.; Ramos, M.; Olson, A. AutoDock4$_{Zn}$: An Improved AutoDock Force Field for Small-Molecule Docking to Zinc Metalloproteins. *J. Chem. Inf. Model.* **2014**, *54*, 2371–2379. PMID: 24931227.
10. Trott, O.; Olson, A. AutoDock Vina: Improving the speed and accuracy of docking with a new scoring function, efficient optimization, and multithreading. *J. Comput. Chem.* **2010**, *31*, 455–461.

11. Amaro, R.; Baudry, J.; Chodera, J.; Demir, Ö.; McCammon, J.; Miao, Y.; Smith, J. Ensemble Docking in Drug Discovery. *Biophys. J.* **2018**, *114*, 2271–2278.
12. Hess, B.; Kutzner, C.; van der Spoel, D.; Lindahl, E. GROMACS 4: Algorithms for Highly Efficient, Load-Balanced, and Scalable Molecular Simulation. *J. Chem. Theory Comput.* **2015**, *4*, 435–447. PMID: 26620784.
13. Maier, J.A.; Martinez, C.; Kasavajhala, K.; Wickstrom, L.; Hauser, K.E.; Simmerling, C. ff14SB: Improving the Accuracy of Protein Side Chain and Backbone Parameters from ff99SB. *J. Chem. Theory Comput.* **2015**, *11*, 3696–3713. PMID: 26574453.
14. Ivani, I.; Dans, P.D.; Noy, A.; Pérez, A.; Faustino, I.; Hospital, A.; Walther, J.; Andrio, P.; Goñi, R.; Balaceanu, A.; Portella, G.; Battistini, F.; Gelpí, J.; González, C.; Vendruscolo, M.; Laughton, C.; Harris, S.; Case, D.; Orozco, M. Parmbsc1: a refined force field for DNA simulations. *Nat. Methods* **2016**, *13*, 55.
15. Jorgensen, W.; Chandrasekhar, J.; Madura, J.; Impey, R.; Klein, M. Comparison of simple potential functions for simulating liquid water. *J. Chem. Theory Comput.* **1983**, *79*, 926–935.
16. Wang, J.; Wolf, R.; Caldwell, J.; Kollman, P.; Case, D. Development and testing of a general amber force field. *J. Comput. Chem.* **2004**, *25*, 1157–1174.
17. Frisch, M.; Trucks, G.; Schlegel, H.; Scuseria, G.; Robb, M.; Cheeseman, J.; Scalmani, G.; Barone, V.; Mennucci, B.; Petersson, G.; Nakatsuji, H.; Caricato, M.; Li, X.; Hratchian, H.; Izmaylov, A.; Bloino, J.; Zheng, G.; Sonnenberg, J.; Hada, M.; Ehara, M.; Toyota, K.; Fukuda, R.; Hasegawa, J.; Ishida, M.; Nakajima, T.; Honda, Y.; Kitao, O.; Nakai, H.; Vreven, T.; Montgomery, J.; Peralta, J.; Ogliaro, F.; Bearpark, M.; Heyd, J.; Brothers, E.; Kudin, K.; Staroverov, V.; Kobayashi, R.; Normand, J.; Raghavachari, K.; Rendell, A.; Burant, J.; Iyengar, S.; Tomasi, J.; Cossi, M.; Rega, N.; Millam, J.; Klene, M.; Knox, J.; Cross, J.; Bakken, V.; Adamo, C.; Jaramillo, J.; Gomperts, R.; Stratmann, R.; Yazyev, O.; Austin, A.; Cammi, R.; Pomelli, C.; Ochterski, J.; Martin, R.; Morokuma, K.; Zakrzewski, V.; Voth, G.; Salvador, P.; Dannenberg, J.; Dapprich, S.; Daniels, A.; Farkas, Ö.; Foresman, J.; Ortiz, J.; Cioslowski, J.; Fox, D. Gaussian 09 Revision E.01, 2009. Gaussian Inc. Wallingford CT.
18. Bayly, C.; Cieplak, P.; Cornell, W.; Kollman, P. A well-behaved electrostatic potential based method using charge restraints for deriving atomic charges: the RESP model. *J. Phys. Chem.* **1993**, *97*, 10269–10280.
19. Cornell, W.; Cieplak, P.; Bayly, C.; Kollman, P. Application of RESP charges to calculate conformational energies, hydrogen bond energies, and free energies of solvation. *J. Phys. Chem.* **1993**, *115*, 9620–9631.
20. Wang, J.; Wang, W.; Kollman, P.; Case, D. Automatic atom type and bond type perception in molecular mechanical calculations. *J. Mol. Graphics Modell.* **2006**, *25*, 247–260.
21. Case, D.; Ben-Shalom, I.; Brozell, S.; Cerutti, D.; Cheatham, T.; Cruzeiro, III, V.; Darden, T.; Duke, R.; Ghoreishi, D.; Giambasu, G.; Giese, T.; Gilson, M.; Gohlke, H.; Goetz, A.; Greene, D.; Harris, R.; Homeyer, N.; Huang, Y.; Izadi, S.; Kovalenko, A.; Krasny, R.; Kurtzman, T.; Lee, T.; LeGrand, S.; Li, P.; Lin, C.; Liu, J.; Luchko, T.; Luo, R.; Man, V.; Mermelstein, D.; Merz, K.; Miao, Y.; Monard, G.; Nguyen, C.; Nguyen, H.; Onufriev, A.; Pan, F.; Qi, R.; Roe, D.; Roitberg, A.; Sagui, C.; Schott-Verdugo, S.; Shen, J.; Simmerling, C.; Smith, J.; Swails, J.; Walker, R.; Wang, J.; Wei, H.; Wilson, L.; Wolf, R.; Wu, X.; Xiao, L.; Xiong, Y.; York, D.; Kollman, P. Amber 2019, 2019. University of California, San Francisco.
22. Sousa da Silva, A.; Vranken, W. ACPYPE – AnteChamber PYthon Parser interfacE. *BMC Res. Notes* **2012**, *5*, 367.
23. Nosé, S. Molecular-Dynamics Method for Simulations in the Canonical Ensemble. *Mol. Phys.* **1984**, *52*, 255–268.
24. Hoover, W. Canonical Dynamics – Equilibrium Phase-Space Distributions. *Phys. Rev. A* **1985**, *31*, 1695–1697.
25. Parrinello, M.; Rahman, A. Polymorphic Transitions in Single-Crystals - a New Molecular-Dynamics Method. *Mol. Phys.* **1981**, *52*, 7182–7190.
26. Darden, T.; York, D.; Pedersen, L. Particle Mesh Ewald - an N.Log(N) Method for Ewald Sums in Large Systems. *J. Chem. Phys.* **1993**, *98*, 10089–10092.
27. Essmann, U.; Perera, L.; Berkowitz, M. A Smooth Particle Mesh Ewald Method. *J. Chem. Phys.* **1995**, *103*, 8577–8593.
28. Hess, B.; Bekker, H.; Berendsen, H.; Fraaije, J. LINCS: A linear constraint solver for molecular simulations. *J. Comput. Chem.* **1997**, *18*, 1463–1472.
29. Daura, X.; Gademann, K.; Jaun, B.; Seebach, D.; van Gunsteren, W.; Mark, A.E. Peptide Folding: When Simulation Meets Experiment. *Angew. Chem. Int. Ed.* **1999**, *38*, 236–240.

30. The PyMOL Molecular Graphics System, Version 1.8, 2015. Schrödinger, LLC.
31. Sander, T.; Freyss, J.; von Korff, M.; Rufener, C. DataWarrior: An Open-Source Program For Chemistry Aware Data Visualization And Analysis. *J. Chem. Inf. Model.* **2015**, *55*, 460–473. PMID: 25558886.
32. Krause, D.; Thörnig, P. JURECA: Modular supercomputer at Jülich Supercomputing Centre. *JLSRF* **2018**, *4*, A132.

© 2020 by the authors. Submitted to *Molecules* for possible open access publication under the terms and conditions of the Creative Commons Attribution (CC BY) license (http://creativecommons.org/licenses/by/4.0/).
